# Comparative study on the biodegradability of morpholinium herbicidal ionic liquids

**DOI:** 10.1007/s10532-015-9737-2

**Published:** 2015-06-23

**Authors:** Łukasz Ławniczak, Katarzyna Materna, Grzegorz Framski, Alicja Szulc, Anna Syguda

**Affiliations:** Faculty of Chemical Technology, Poznan University of Technology, Berdychowo 4, 60-965 Poznań, Poland; Institute of Bioorganic Chemistry, Polish Academy of Sciences, Noskowskiego 12/14, 61-704 Poznań, Poland

**Keywords:** Biodegradation, Ionic liquids, Herbicides, Surface active properties, Toxicity

## Abstract

This study focused on evaluating the toxicity as well as primary and ultimate biodegradability of morpholinium herbicidal ionic liquids (HILs), which incorporated MCPA, MCPP, 2,4-D or Dicamba anions. The studied HILs were also subjected to determination of surface active properties in order to assess their influence on toxicity and biodegradability. The study was carried out with microbiota isolated from different environmental niches: sediments from river channel, garden soil, drainage trench collecting agricultural runoff stream, agricultural soil and municipal waste repository. The obtained results revealed that resistance to toxicity and biodegradation efficiency of the microbiota increased in the following order: microbiota from the waste repository > microbiota from agricultural soil ≈ microbiota from an agricultural runoff stream > microbiota from garden soil > microbiota from the river sludge. It was observed that the toxicity of HILs increased with the hydrophobicity of the cation, however the influence of the anion was more notable. The highest toxicity was observed when MCPA was used as the anion (EC_50_ values ranging from 60 to 190 mg L^−1^). The results of ultimate biodegradation tests indicated that only HILs with 2,4-D as the anion were mineralized to some extent, with slightly higher values for HILs with the 4-decyl-4-ethylmorpholinium cation (10–31 %) compared to HILs with the 4,4-didecylmorpholinium cation (9–20 %). Overall, the cations were more susceptible (41–94 %) to primary biodegradation compared to anions (0–61 %). The obtained results suggested that the surface active properties of the studied HILs may influence their toxicity and biodegradability by bacteria in different environmental niches.

## Introduction

Herbicidal ionic liquids (HILs) are defined as organic salts containing herbicidal anions with a melting point below 100 °C (Pernak et al. 2011). The idea behind this group of compounds is focused on taking advantage of their limited volatility, while enhancing their efficiency (the weed killing effect is obtained at lower concentrations) or adding other valuable properties e.g., increased wettability, which would be responsible for enhanced uptake by the plants (Pernak et al. 2013a; Niemczak et al. 2015). Several recent reports confirm, that herbicides such as 2,4-D (Pernak et al. 2012, Praczyk et al. 2012), MCPA (Kordala-Markiewicz et al. 2014), Dicamba (Cojocaru et al. 2013), Glyphosate (Pernak et al. 2014a), Fomesafen (Ding et al. 2014) or Sulfonylurea (Pernak et al. 2015a) can be modified by transforming them into corresponding HILs. Additionally, HILs with dual pesticidal function, which exhibit both herbicidal and fungicidal activity, have also been introduced (Pernak et al. 2013b, 2014b). In this study, two derivatives of morpholine were selected as cations. This water soluble amino ether is commonly applied in agrochemicals, biocides and fungicides, partly because of its surface active properties (Lamberth 2012). The formation of the ionic liquid structure may notably change the physicochemical properties of the original pesticide (Pernak et al. 2015b). Therefore incorporation of a surface active cation in HILs may potentially result in the alteration of their behaviour in the biotope and their influence on the ecosystem. However, it should be noted that since HILs are relatively new, there is little data regarding their impact on the environment. Such information is essential, since these compounds might be introduced directly into the environment and negatively influence both biotic (i.e. biotransformation into harmful metabolites resulting in toxicity towards plant and animal species, bioaccumulation caused by food chain dependency) and abiotic factors (i.e. prolonged contamination due to sorption, interactions with soil components resulting in changes of soil characteristics and properties). It is therefore imperative to study their toxicity and biodegradability to ascertain whether these compounds exhibit the potential to accumulate in the environment.

The main aim of this study was focused on investigating the ultimate and primary biodegradability of several morpholine-based HILs with respect to microorganisms isolated from different environmental niches: river sludge, garden soil, agricultural run-off streams, agricultural soil and waste repository. The secondary aim was to evaluate the influence of surface active properties of the studied HILs (CMC, surface tension, contact angle (CA), etc.) on their toxicity and biodegradability.

## Materials and methods

### Preparation of herbicidal ionic liquids

**Fig. 1:**
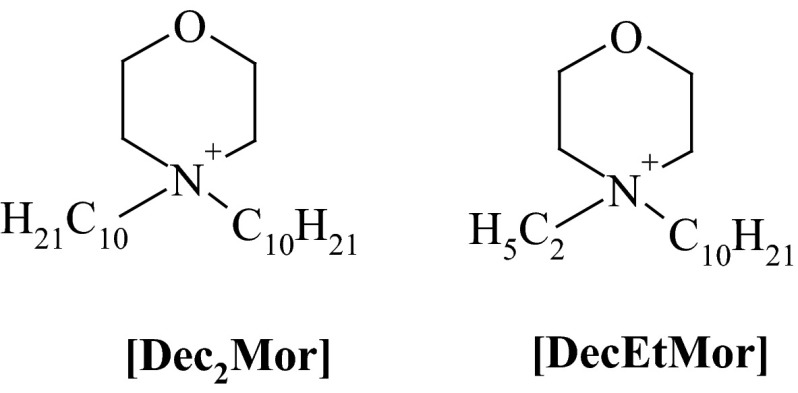
Structures of cations used for the synthesis of the studied HILs

**Fig. 2:**
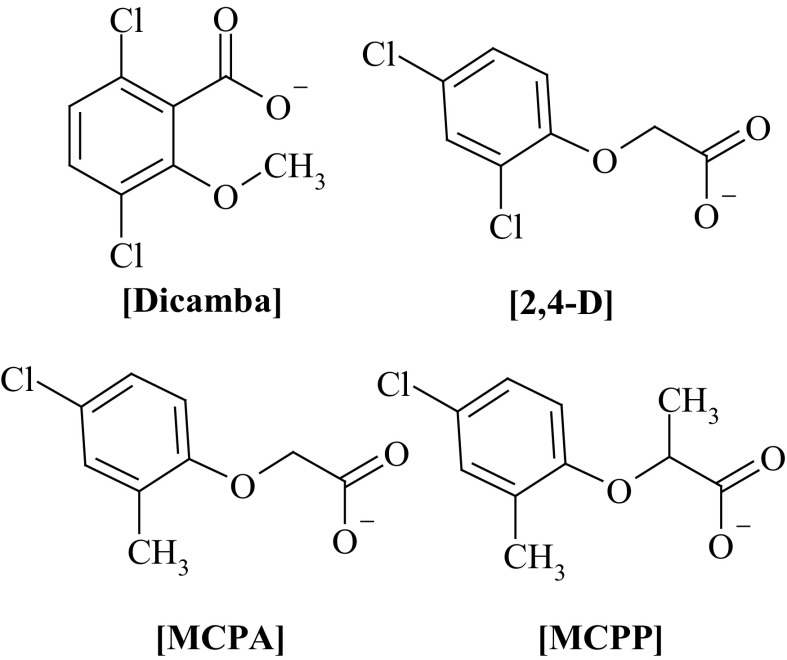
Structures of anions used for the synthesis of the studied HILs

The chemical structures of cations and anions used for the synthesis of the studied HILs were presented in Figs. 1 and 2, accordingly.

#### 4-Decylmorpholine

Morpholine (0.25 mol) was dissolved in toluene (100 mL) and stirred with a stoichiometric amount of decyl bromide and 0.3 mol of NaHCO_3_. The reacting vessel was heated to boiling temperature under a reflux condenser for 24 h. The produced NaBr was filtered off and washed by toluene, then the toluene from the organic phase was removed by evaporation. Finally, the product was distilled under reduced pressure (boiling point 136 °C at 7 hPa). The yield of 4–decylmorpholine preparation was 86 %.

#### 4,4-Dialkylmorpholinium bromides

Decylbromide (0.1 mol) was added into a round-bottomed flask which contained a vigorously stirred mixture of 50 mL acetonitrile and 0.1 mol of 4-decyl- or 4-ethylmorpholine (purchased from Fluka, purity >97 %). The reaction mixture was stirred at boiling temperature under a reflux condenser for 48 h. Afterwards acetonitrile was removed under reduced pressure and 20 mL of ethyl acetate was added. The precipitate was filtered, washed with hexane and dried at 60 °C under reduced pressure. The yield of 4-decyl-4-ethylmorpholinium bromide preparation was 94 % (melting point at 191–192 °C) and the surfactant content was at 95 %. The yield of 4,4-didecylmorpholinium bromide was 68 % (melting point at 148–150 °C), and the surfactant content was at 98 %.

#### Ionic liquids

The reagent mixture consisting of 0.01 mol of the selected herbicide in the acid form (Dicamba, 2,4-D, MCPA or MCPP), 20 mL of distilled water and 0.011 mol of 10 % aqueous solution of NaOH was mixed in a round-bottomed flask, equipped with a magnetic stirring bar, a reflux condenser and an addition funnel. The mixture was heated at 50 °C until the solution became clear. Afterwards, 0.01 mol of 4,4-didecylmorpholinium ([Dec_2_Mor]) or 4-decyl-4-ethylmorpholinium ([DecEtMor]) bromide dissolved in 30 mL of water was added and stirred for 30 min at room temperature. In the case of 4,4-didecylmorpholinium-based HILs, the product was extracted from the aqueous phase with 50 mL of chloroform and washed with distilled water until bromide ions were no longer detected using AgNO_3_. After removal of chloroform, the product was dried under reduced pressure at 60 °C for 24 h. In the case of 4-decyl-4-ethylmorpholinium-based HILs, the water was removed under reduced pressure until constant weight. Anhydrous acetone was added to dissolve the product, the liquid was then filtered and acetone was evaporated. The product was dried under reduced pressure at 60 °C for 24 h. The yields and surfactant content for each obtained HIL were presented in Table 1.

**Table 1:** Basic properties of the obtained HILs

Acronym	Surfactant content (%)	Yield (%)	State at 25 °C
[Dec_2_Mor][Dicamba]	98.5	98	Grease
[Dec_2_Mor][2,4-D]	98.0	90	Grease
[Dec_2_Mor][MAPA]	98.0	96	Grease
[Dec_2_Mor][MCPP]	99.5	92	Grease
[DecEtMor][Dicamba]	99.0	90	Liquid
[DecEtMor][2,4-D]	98.0	91	Liquid
[DecEtMor][MAPA]	98.0	94	Liquid
[DecEtMor][MCPP]	98.5	93	Liquid

ISO 2871—1:1988. “Surface active agents—Detergents—Determination of cationic-active matter content—Part 1: High-molecular mass cationic-active matter”, ISO 2871—2:1990. “Surface active agents—Detergents—Determination of cationic-active matter content—Part 2: Cationic-active matter of low molecular mass (between 200 and 500)”

^1^H NMR spectra were recorded on a Varian VNMR-S spectrometer at 400 MHz with tetramethylsilane as the standard; ^13^C NMR spectra were recorded on the same instrument at 100 MHz. Elemental analyses were performed at Adam Mickiewicz University, Poznan.

#### 4,4-Didecylmorpholinium 3,6-dichloro-2-methoxybenzoate

[Dec_2_Mor][Dicamba]^1^H NMR (CDCl_3_) δ ppm = 0.88 (t, *J* = 6.7 Hz, 6H), 1.26 (m, 28H), 1.76 (q, *J* = 4.5 Hz, 4H), 2.82 (t, *J* = 8.3 Hz, 4H) 3.00 (t, *J* = 4.7 Hz, 4H), 3.94 (s, 3H), 3.98 (t, *J* = 4.9 Hz, 4H), 7.05 (d, *J* = 8.6 Hz, 1H), 7.17 (d, *J* = 8.6 Hz, 1H); ^13^C NMR δ ppm = 14.0, 22.5, 23.9, 26.9, 29.1, 29.3, 29.4, 31.7, 51.8, 57.8, 61.7, 64.5, 125.5, 126.2, 128.2, 128.7, 136.7, 152.2, 169.6. Anal. Calcd for C_32_H_55_O_4_NCl_2_: C 65.27, H 9.43, N 2.38; Found: C 65.69 1, H 9.12, N 2.12.

#### 4,4-Didecylmorpholinium 2,4-dichlorophenoxyacetate

[Dec_2_Mor][2,4-D]^1^H NMR (CDCl_3_) δ ppm = 0.88 (t, *J* = 6.9 Hz, 6H), 1.26 (m, 28H), 1.62 (q, *J* = 5.9 Hz, 4H), 3.49 (t, *J* = 5.3 Hz, 4H), 3.65 (t, *J* = 4.6 Hz, 4H), 3.94 (t, *J* = 4.5 Hz, 4H), 4.46 (s, 2H), 6.91 (d, *J* = 8.9 Hz, 1H), 7.10 (dd, *J*^1,2^ = 2.7 Hz, *J*^1,3^ = 8.8 Hz, 1H) 7.29 (d, *J* = 2.6 Hz, 1H); ^13^C NMR δ ppm = 13.9, 21.3, 22.4, 26.1, 29.0, 29.2, 31.6, 53.6, 57.8, 60.2, 69.1, 114.7, 122.3, 124.4, 127.4, 129.1, 153.7, 171.5. Anal. Calcd for C_32_H_55_O_4_NCl_2_: C 65.27, H 9.43, N 2.38; Found: C 65.00 1, H 9.63, N 2.49.

#### 4,4-Didecylmorpholinium 4-chloro-2-methylphenoxyacetate

[Dec_2_Mor][MCPA]^1^H NMR (CDCl_3_) δ ppm = 0.88 (t, *J* = 6.8 Hz, 6H), 1.26 (m, 28H), 1.61 (q, *J* = 5.9 Hz, 4H), 2.24 (s, 3H), 3.48 (t, *J* = 8.6 Hz, 4H) 3.62 (t, *J* = 4.9 Hz, 4H), 3.96 (t, *J* = 4.9 Hz, 4H), 4.43 (s, 2H), 6.75 (d, *J* = 8.6 Hz, 1H), 7.00 (dd, *J*^1,2^ = 2.8 Hz, *J*^1,3^ = 8.6 Hz, 1H) 7.04 (d, *J* = 2.8 Hz, 1H); ^13^C NMR δ ppm = 13.9, 16.2, 21.4, 22.4, 26.1, 29.0, 29.1, 31.6, 53.5, 57.8, 60.2, 66.7, 112.8, 124.0, 125.9, 128.3, 129.8, 156.1, 172.6. Anal. Calcd for C_33_H_58_O_4_NCl_2_: C 69.73, H 10.31, N 2.46; Found: C 69.38, H 10.12, N 2.31.

#### 4,4-Didecylmorpholinium (±)-2-(4-chloro-2-methylphenoxy)propionate

[Dec_2_Mor][MCPP]^1^H NMR (CDCl_3_) δ ppm = 0.89 (t, *J* = 6.7 Hz, 6H), 1.27 (m, 28H), 1.57 (d, *J* = 6.9 Hz, 3H), 1.60 (q, *J* = 7.2 Hz, 4H), 2.23 (s, 3H), 3.43 (t, *J* = 8.5 Hz, 4H) 3.56 (t, *J* = 4.9 Hz, 4H), 3.91 (t, *J* = 4.9 Hz, 4H), 4.45 (qua, *J* = 6.9 Hz, 1H), 6.79 (d, *J* = 8.7 Hz, 1H), 6.96 (dd, *J*^1,2^ = 2.7 Hz, *J*^1,3^ = 7.7 Hz, 1H) 7.03 (d, *J* = 2.7 Hz, 1H);^13^C NMR δ ppm = 13.9, 16.3, 19.3, 21.4, 22.5, 26.1, 26.3, 29.0, 29.2, 31.6, 53.5, 57.6, 60.2, 66.7, 113.4, 123.6, 125.8, 129.7, 155.9, 176.1. Anal. Calcd for C_34_H_60_O_4_NCl_2_: C 70.11, H 10.41, N 2.41; Found: C 70.49, H 10.22, N 2.56.

#### 4-Decyl-4-ethylmorpholinium 3,6-dichloro-2-methoxybenzoate

[DecEtMor][Dicamba]^1^H NMR (D_2_O) δ ppm = 0.98 (t, *J* = 6.0 Hz, 3H), 1.13 (m, 14H), 1.21 (t, *J* = 6.0 Hz, 3H), 1.49 (q, *J* = 4.0 Hz, 2H) 3.22 (t, *J* = 4.4 Hz, 2H), 3.44 (t, *J* = 4.4 Hz, 2H), 3.49 (t, *J* = 4.4 Hz, 4H), 3.86 (s, 3H), 3.99 (t, *J* = 4.9 Hz, 4H), 7.07 (d, *J* = 8.6 Hz, 1H), 7.12 (d, *J* = 8.6 Hz, 1H); ^13^C NMR δ ppm = 8.7, 16.1, 23.1, 24.9, 28.3, 31.3, 31.7, 31.8, 34.2, 55.9, 59.7, 60.5, 62.3, 64.0, 127.7, 128.0, 130.1, 130.3, 140.3, 153.9, 171.9. Anal. Calcd for C_24_H_39_O_4_NCl_2_: C 60.49, H 8.27, N 2.94; Found: C 60.01, H 8.00, N 2.79.

#### 4-Decyl-4-ethylmorpholinium 2,4-dichlorophenoxyacetate

[DecEtMor][2,4-D]^1^H NMR (D_2_O) δ ppm = 0.97 (t, *J* = 6.9 Hz, 3H), 1.20 (m, 14H), 1.34 (t, *J* = 6.9 Hz, 3H), 1.52 (q, *J* = 4.0 Hz, 2H) 3.20 (t, *J* = 8.1 Hz, 2H), 3.38 (t, *J* = 4.9 Hz, 2H), 3.45 (t, *J* = 4.4 Hz, 4H), 4,02 (t, *J* = 4.9 Hz, 4H), 4.84 (s, 2H), 6.94 (d, *J* = 9,2 Hz, 1H), 7.20 (dd, *J*^1,2^ = 2.6 Hz, *J*^1,3^ = 9.0 Hz, 1H) 7.22 (d, *J* = 2.6 Hz, 1H); ^13^C NMR δ ppm = 8.8, 16.3, 25.1, 28.5, 31.6, 31.9, 34.4, 56.2, 59.9, 60.5, 62.4, 70.3.0, 117.2, 124.8, 127.4, 130.4, 131.6, 155.4, 176.0. Anal. Calcd for C_24_H_39_O_4_NCl_2_: C 60.49, H 8.27, N 2.94; Found: C 60.88, H 8.48, N 3.11.

#### 4-Decyl-4-ethylmorpholinium 4-chloro-2-methylphenoxyacetate

[DecEtMor][MCPA]^1^H NMR (D_2_O) δ ppm = 0.97 (t, *J* = 7.2 Hz, 3H), 1.22 (m, 14H), 1.29 (t, *J* = 6.8 Hz, 3H), 1.46 (q, *J* = 8.5 Hz, 2H) 2.22 (s, 3H), 3.12 (t, *J* = 8.6 Hz, 2H), 3.26 (t, *J* = 3.6 Hz, 2H), 3.34 (t, *J* = 7.1 Hz, 4H), 3.93 (t, *J* = 4.9 Hz, 4H), 4,82 (s, 2H), 6.76 (d, *J* = 8.8 Hz, 1H), 7.09 (dd, *J*^1,2^ = 2.8 Hz, *J*^1,3^ = 8.7 Hz, 1H) 7.01 (d, *J* = 2.8 Hz, 1H); ^13^C NMR δ ppm = 8.8, 16.3, 18.5, 23.2, 25.1, 28.5, 31.6, 31.9, 34.4, 55.9, 59.9, 60.6, 62.4, 69.8, 115.1, 126.8, 128.9, 131.1, 132.4, 157.8, 177.3. Anal. Calcd for C_25_H_42_O_4_NCl: C 65.83, H 9.30, N 3.07; Found: C 65.48, H 9.14, N 3.29.

#### 4-Decyl-4-ethylmorpholinium (±)-2-(4-chloro-2-methylphenoxy)propionate

[DecEtMor][MCPP]^1^H NMR (D_2_O) δ ppm = 0.96 (t, *J* = 7.0 Hz, 3H), 1.29 (m, 14H), 1.35 (t, *J* = 6,8 Hz, 3H), 1.46 (q, *J* = 8.5 Hz, 2H), 1.52 (d, *J* = 6.7 Hz, 3H), 2.22 (s, 3H), 3.10 (t, *J* = 8.6 Hz, 2H), 3.23 (t, *J* = 5.4 Hz, 2H), 3.31 (t, *J* = 6.3 Hz, 4H), 3.90 (t, *J* = 4.9 Hz, 4H), 4,43 (qua, *J* = 6.9 Hz, 1H), 6.71 (d, *J* = 8.9 Hz, 1H), 7.01 (dd, *J*^1,2^ = 2.7 Hz, *J*^1,3^ = 8.9 Hz, 1H) 7.09 (d, *J* = 2.7 Hz, 1H); ^13^C NMR δ ppm = 8.9, 16.3, 18.6, 21.1, 23.2, 25.1, 28.5, 31.5, 31.9, 32.0, 32.1, 34.4, 56.1, 59.9, 60.5, 62.4, 78.0, 115.8, 126.5, 128.8, 131.4, 132.3, 157.8, 181.2. Anal. Calcd for C_26_H_44_O_4_NCl: C 66.42, H 9.45, N 2.98; Found: C 66.78, H 8.66, N 3.05.

### Sample collection

The environmental samples have been collected from the following sites: sediments from river Warta channel (Poznań), garden soil (land plot near Poznań), drainage trench collecting agricultural runoff stream (Winna Góra), agricultural soil (Winna Góra) and municipal waste repository (Poznań). The sediments and runoff samples were collected according to the procedure described by Kang and Kondo (2002), whereas the soil samples were collected according to the procedure described by Ito et al. (2008).

### Determination of surface active properties of the studied HILs

Surface tension and CA measurements were carried out by the use of a Drop Shape Analysis System DSA100E (KRÜSS GmbH, Germany, accuracy ±0.01 mN m^−1^), at 25 °C. Temperature was controlled using a Fisherbrand FBH604 thermostatic bath (Fisher, Germany, accuracy ±0.1 °C). The surface tension was determined using the pendant drop method. This method consists of fitting the Young–Laplace equation to the digitized shape of a drop suspended from the end of a capillary tube. The image of the drop (6 μL) was taken from a charge coupled device (CCD) camera. The values of the critical micelle concentration (CMC) and the surface tension at the CMC (γCMC) were determined from the intersection of the two straight lines drawn in low and high concentration regions in surface tension curves (γ vs log C curves) using a linear regression analysis method.

The CA was measured using the sessile drop method (Young–Laplace), i.e. drop of liquid was deposited on a solid surface (paraffin). The drop was produced before the measurement and had a constant volume during the measurement. In this method the complete drop contour was evaluated. After the successful fitting of the Young–Laplace equation the CA was determined as the slope of the contour line at the 3-phase contact point.

### Toxicity studies

#### Preparation of cultures for toxicity studies

In all cases the enrichment technique was performed by placing 5 g or 5 mL of appropriate sample material (soil, sediment, etc.) into a 250 mL-Duran bottle containing 50 mL of mineral salt medium (MSM, composition given in Rousseaux et al. 2001) and glucose as a carbon source (5 g L^−1^). Supplementation with glucose is often suggested as means to obtain maximum microbial activity and growth during toxicity testing protocols (OECD 2000). Cultures were incubated at 30 °C and 120 rpm on a rotary shaker for 7 days. In the next step 5 mL of the enrichment culture were transferred into Duran bottle containing 50 mL of fresh MSM media and glucose. Cultures were incubated at 30 °C and 120 rpm on a rotary shaker for 7 days. The enrichment step was repeated every 7 days in order to obtain pure biomass and eliminate any remains of the environmental matrix (particulate matter, etc.). Prior to the determination of toxicity, 1 mL aliquot of the last enrichment was transferred to a new Duran bottle and the culture was grown for 3 days in the same conditions. The cells were centrifuged at 10,000×*g*, washed twice with 5 mL of MSM and re-suspended. The final cultures were used to inoculate the experimental samples. Aerobic conditions were provided during all steps. After the enrichment procedure of the microbiota stock, the cultures were set up to an OD600 of 0.1 ± 0.01 for each experimental condition.

#### Determination of toxicity of the studied HILs

The toxicity of the synthesized HILs and reference compounds (4,4-Dialkylmorpholinium bromides, 2,4-D, MCPA, MCPP and Dicamba) towards isolated microbiota was tested according to the procedure described by Wyrwas et al. (2013). The isolates were grown in 250 mL-Duran bottles containing 50 mL of MSM and glucose (5 g L^−1^). The toxicity was quantified by the effect of the studied compounds on the microbial growth rates. Stock solutions of HILs in methanol were prepared and afterwards 1 mL of the corresponding solution was added to exponentially growing cells in order to achieve the following concentrations: 1, 2.5, 10, 25, 50, 100, 250 and 500 mg L^−1^. A control sample containing 50 mL of MSM, glucose (5 g L^−1^) and 1 mL of methanol (culture without the studied compounds) was prepared for comparison. The growth inhibition was defined as the percentage of growth rates of cultures with compounds of interest and that of control. Effective concentrations of the compounds causing 50 % growth inhibition were expressed as EC50 values. All samples were prepared in triplicates.

### Biodegradation studies

#### Preparation of cultures for biodegradation studies

Isolation of microbiota capable of biodegrading the studied HILs from each environmental niche was based on the procedure described by Batisson et al. (2009). Enrichment cultures were prepared by placing 5 g of appropriate sample material (soil, sediment, etc.) into a 250 mL-Duran bottle containing 50 mL of MSM. Stock solutions of HILs as well as reference compounds (4,4-Dialkylmorpholinium bromides, 2,4-D, MCPA, MCPP and Dicamba) in methanol were prepared and afterwards 1 mL of the corresponding solution was added to appropriate cultures in order to achieve a concentration of 25 mg L^−1^. After 3 weeks a fresh enrichment culture was inoculated with 1 mL of the precedent culture and the concentration of the target compound was doubled. This step was repeated three times in order to reach the maximum concentration of 100 mg L^−1^ in the final culture. Cultures were incubated at 28 °C and 150 rpm on a rotary shaker. No remains of the environmental matrix (particulate matter, etc.) were present in the last enrichment culture due to successive dilutions. The cells were centrifuged at 10,000×*g*, washed twice with 5 mL of MSM and re-suspended. The final cultures were used to inoculate subsequent experimental samples. Aerobic conditions were provided during all steps. After the enrichment procedure of the microbiota stock, the cultures were set up to an OD600 of 0.1 ± 0.01 for each experimental condition.

#### Evaluation of ultimate biodegradation of the studied HILs

The ultimate biodegradability of the studied HILs was evaluated based on the OECD 301 F test (manometric respirometry), which was conducted with a single modification: the tests were carried out with the use of microbiota previously isolated from environmental niches (as described in 2.4.1) instead of the routinely applied activated sludge. The subsequent testing procedures were carried out according to the OECD protocol. The biological oxygen demand (BOD) was determined every 24 h for 28 days using an OxiTop system (WTW GmbH Weilheim Germany) in a thermostated incubator (IKA Germany) covered with aluminium foil. The pH was at 7.2. The test was performed in brown glass bottles containing MSM, inoculum (cell density at approximately 10^6^ cells mL^−1^ determined with plastic Paddle Tester for aerobic bacteria, Hach, USA) and tested substances at a concentration of approximately 10–30 mg L^−1^, which was equal to 100 mg L^−1^ of theoretical oxygen demand (ThOD). Allylthiourea (1.16 mg L^−1^) was added to inhibit nitrification. All samples were analysed in triplicates together with controls (sodium benzoate without inoculum, tested substances without inoculum) and blank (MSM and inoculum without tested substances). Gas tight flasks were equipped with a CO_2_ trap (solid NaOH) and incubated in the dark at 20 °C for 28 days. The biodegradation efficiency was calculated based on the oxygen uptake in each bottle (measured automatically by the electronic OxiTop head) and corrected for the oxygen demand of the blank and with the respect to the ThOD (calculated based on Eq. 1) and the amount of substance tested.

Equation 1. ThOD for a chemical formula given as C_c_H_h_N_n_O_o_P_p_:

1 $${\text{ThOD}} = \frac{{16\left[ {{\text{2c}} + \frac{1}{2}\left( {{\text{h}} - 3{\text{n}}} \right) + \frac{5}{2}{\text{p}} - {\text{o}}} \right]\frac{\text{mg}}{\text{mg}}}}{{{\text{molecular mass of the test substance }}\frac{\text{mmol}}{\text{mg}}}}$$

In case of HIL precursors (bromides) the ThOD value was calculated for the cations without including the inorganic anions.

#### Evaluation of primary biodegradation of the studied HILs

The evaluation of primary biodegradation efficiency was carried out according to the HPLC–MS analysis procedure described in Pernak et al. (2014b).

### Statistical analysis

Each error margin range represent standard errors of the mean (SEM). The SEM values were calculated according to Eq. 2.

Equation 2. Calculation of SEM values.

2 $$SEM = \frac{s}{{n^{0.5} }}$$

where *SEM* standard error of the mean , *s* sample standard deviation, *n* number of samples.Analyses of variance at α = 0.05 were performed in order to determine the significance of test results (one-way ANOVA).

## Results and discussion

### Determination of surface active properties of the studied HILs

The first experimental step was focused on evaluating the surface active properties of the studied HILs. The obtained results were shown in Table 2. The values represent CMC, the surface tension of the solution at the CMC (γCMC), the negative logarithm of the surfactant concentration in the bulk phase required to reduce the surface tension of the water by 20 mN m^−1^, which represents efficiency of surface adsorption on an air–water interface (pC_20_), the surface pressure at the CMC, which indicates the effectiveness of the surfactant to lower the surface tension of the solvent (Π_CMC_), surface excess concentrations (Γ_max_), minimum area per molecule (A_min_) and CA, accordingly.

**Table 2:** Parameters describing the surface active properties of the studied HILs

Studied HIL	CMC (mol L^−1^)	γ_CMC_ (mN m^−1^)	pC_20_	Π_CMC_ (mN m^−1^)	Γ_max_ (μmol m^−2^)	A_min_ (10^−19^m^2^)	CA (°)
[Dec_2_Mor][MCPA]	3.09 × 10^−4^	27.3	4.60	45.5	5.22	3.18	34.2
[Dec_2_Mor][MCPP]	3.24 × 10^−4^	27.7	4.75	45.1	5.57	2.99	34.4
[Dec_2_Mor][2.4-D]	3.05 × 10^−4^	27.2	4.68	45.6	5.07	3.27	29.9
[Dec_2_Mor][Dicamba]	3.98 × 10^−3^	29.1	3.36	43.7	5.64	2.94	47.4
[DecEtMor][MCPA]	1.02 × 10^−2^	30.2	2.79	42.6	6.67	2.49	53.9
[DecEtMor][MCPP]	0.79 × 10^−2^	29.2	3.09	43.6	8.59	1.93	54.7
[DecEtMor][2.4-D]	1.05 × 10^−2^	32.2	2.80	40.6	6.19	2.68	58.6
[DecEtMor][Dicamba]	1.23 × 10^−2^	35.1	2.49	37.7	10.3	1.62	64.0

The analysis of surface active properties revealed differences related to the structure of the cation. Overall, HILs with the [Dec_2_Mor]^+^ cation exhibited superior surface active properties compared to HILs with the [DecEtMor]^+^ cation. The CMC value of HILs with the [Dec_2_Mor]^+^ cation was one or two orders of magnitude lower and this group of compounds decreased the surface tension more efficiently compared to the corresponding HILs with the [DecEtMor]^+^ cation. The CA value, which reflects the hydrophilic or hydrophobic affinity of the compound, suggested that HILs with the [Dec_2_Mor]^+^ cation displayed more hydrophobic properties (lower CA), as opposed to HILs with the [DecEtMor]^+^ cation, which were generally more hydrophilic (higher CA). The measurement was carried out on paraffin surface, therefore compounds with higher the CA value were more hydrophilic (the CA value for a water droplet is approximately 111*°*). This, as well as the higher minimum area per molecule (A_min_) values for HILs with the [Dec_2_Mor]^+^ cation, can be attributed to the structural difference between the cations, namely the presence of another decyl substituent. The influence of the anion on the surface active properties was marginal, with the sole exception of HILs with the [Dicamba]^−^ anions, which were inferior compared to HILs with other herbicidal anions.

### Determination of toxicity of the studied HILs

The second experimental step was focused on determining the toxicity of the studied HILs towards microbial isolates obtained from different environmental niches. The obtained results are shown in Table 3. The values represent the concentration of a given IL, which caused a 50 % inhibition of microbial growth (EC_50_ value), expressed as mg L^−1^.

**Table 3:** Toxicity of the studied HILs towards microbial isolates obtained from different environmental niches

Studied compound	EC50 value (mg L^−1^)				
	Microbiota from river sludge	Microbiota from garden soil	Microbiota from an agricultural runoff stream	Microbiota from agricultural soil	Microbiota from a waste repository
[Dec_2_Mor][MCPA]	45 ± 12	82 ± 17	98 ± 21	103 ± 16	150 ± 23
[Dec2Mor][MCPP]	60 ± 19	94 ± 26	132 ± 31	144 ± 19	182 ± 30
[Dec2Mor][2,4-D]	104 ± 34	113 ± 31	195 ± 46	211 ± 33	222 ± 19
[Dec_2_Mor][Dicamba]	>500	>500	>500	>500	>500
[DecEtMor][MCPA]	69 ± 21	103 ± 29	115 ± 23	144 ± 45	190 ± 25
[DecEtMor][MCPP]	79 ± 25	131 ± 36	149 ± 19	187 ± 33	208 ± 21
[DecEtMor][2,4-D]	148 ± 38	184 ± 30	227 ± 37	260 ± 26	277 ± 31
[DecEtMor][Dicamba]	>500	>500	>500	>500	>500
[Dec_2_Mor][Br]	65 ± 9	96 ± 14	106 ± 22	134 ± 19	136 ± 13
[DecEtMor][Br]	83 ± 16	140 ± 32	122 ± 9	152 ± 31	182 ± 26
[MCPA]	103 ± 17	122 ± 21	139 ± 17	210 ± 36	245 ± 24
[MCPP]	100 ± 21	138 ± 12	152 ± 24	165 ± 25	175 ± 17
[2,4-D]	193 ± 23	202 ± 27	230 ± 15	211 ± 26	293 ± 42
[Dicamba]	>500	>500	>500	>500	>500

The highest resistance towards the studied HILs was exhibited by microbiota isolated from a waste repository, as shown by the highest EC_50_ values. On the other hand, the microbiota isolated obtained from the river sludge were most susceptible to the toxic effect of the studied HILs, as reflected by the lowest EC_50_ values. In general, the tolerance towards the studied compounds among the HILs may be described by the following order: microbiota from the waste repository > microbiota from agricultural soil > microbiota from an agricultural runoff stream > microbiota from garden soil > microbiota from the river sludge.

The obtained results revealed that the toxicity of the studied HILs was influenced by both the cationic and the anionic moiety used for their synthesis. In general, HILs with the [DecEtMor]^+^ cation were the less toxic (EC_50_ values ranging from 69 to 277 mg L^−1^), whereas HILs with the [Dec_2_Mor]^+^ cation reached the EC_50_ value at lower concentrations (ranging from 45 to 222 mg L^−1^).

Based on the analysis of the influence of the anionic moiety on the toxicity of the studied HILs, it was established that HILs with the [MCPA]^−^ anion were most toxic (EC_50_ values ranging from 45 to 190 mg L^−1^). It was not possible to determine the EC_50_ value for HILs with the [Dicamba]^−^ anion within the studied range of concentrations, which suggests that this anion does not contribute to a toxic effect. The influence of the corresponding anion on the increase of toxicity of the studied HILs can be described by the following order: [MCPA]^−^ > [MCPP]^−^ > [2,4-D]^−^ > [Dicamba]^−^.

The obtained results are in accordance with the reports of other researchers regarding the generally low toxicity of ionic liquids with morpholinium-based cations (Stolte et al. 2007; Cvjetko Bubalo et al. 2014). However, it should also be noted that the use of a more hydrophobic decyl group instead of an ethyl group led to an increase in toxicity, which is similar to the results reported by Pretti et al. (2011). This effect is consistent with the commonly observed trend, that increase in the alkyl chain length of the cation substituent leads to an increase of toxicity (Pham et al. 2010). Comparison of toxicity results with the analysis of surface active properties suggests that in most cases the values for hydrophobic HILs with the [Dec_2_Mor]^+^ cation and hydrophilic HILs with the [DecEtMor]^+^ cation were similar (taking the error margins into account). However, the comparison of results obtained for HILs with those obtained for commercial herbicides indicates an increase of toxicity towards the isolated microbiota, especially for hydrophobic HILs with lower CMC values. On the average, the EC50 values of HILs with the [Dec_2_Mor]^+^ cation were lower by approximately 33 % compared to commercial herbicides in the acid form. The increase of toxicity caused by an increase in the hydrophobicity of the compounds has been reported on several occasions, e.g. for chlorophenols (Chrzanowski et al. 2009, 2011). This effect may be hypothetically explained by the higher affinity of hydrophobic HILs to the cell membrane. The incorporation of surface active compounds to the external layers of cellular structures may contribute to the occurrence of negative phenomena, which disrupt the integrity of the phospholipid bilayer. This may result in uncontrolled bilayer flip-flop, severe malfunction of cellular transport systems and, ultimately, cell lysis. The results obtained for reference compounds revealed that their toxicity was lower compared to that of the corresponding HILs. The EC_50_ values of MCPA and 2,4-D obtained for some of the isolated microbiota were similar to those reported by Sanchis et al. (2014) for experiments with activated sludge (144 and 213 mg L^−1^, accordingly). Additionally, no apparent toxicity of Dicamba observed in the tested concentration range is in accordance with the observations of Drzewicz et al. (2005).

### Evaluation of ultimate biodegradation of the studied HILs

The evaluation of ultimate biodegradation of the studied HILs based on the results of OECD 301 manometric respiration tests was shown in Table 4. The values represent the mineralization efficiency of a given HIL, which was calculated based on the amount of oxygen consumed during the mineralization process, expressed as a percentage relative to the ThOD needed for a complete mineralization of a given compound.

**Table 4:** Results of ultimate biodegradation tests for the studied HILs

Studied compound	Ultimate biodegradation (%)				
	Microbiota from river sludge	Microbiota from garden soil	Microbiota from an agricultural runoff stream	Microbiota from agricultural soil	Microbiota from a waste repository
[Dec_2_Mor][MCPA]	0 ± 0	3 ± 1	3 ± 1	2 ± 1	4 ± 1
[Dec2Mor][MCPP]	0 ± 0	2 ± 1	2 ± 1	2 ± 1	3 ± 1
[Dec2Mor][2,4-D]	9 ± 2	13 ± 3	18 ± 3	14 ± 2	20 ± 3
[Dec_2_Mor][Dicamba]	0 ± 0	0 ± 0	1 ± 1	2 ± 1	2 ± 1
[DecEtMor][MCPA]	0 ± 0	4 ± 1	6 ± 2	5 ± 1	7 ± 1
[DecEtMor][MCPP]	0 ± 0	3 ± 1	3 ± 1	2 ± 1	3 ± 1
[DecEtMor][2,4-D]	10 ± 3	19 ± 3	25 ± 4	24 ± 3	31 ± 3
[DecEtMor][Dicamba]	0 ± 0	1 ± 1	2 ± 1	2 ± 1	2 ± 1
[Dec_2_Mor][Br]	0 ± 0	0 ± 0	0 ± 0	1 ± 1	1 ± 1
[DecEtMor][Br]	0 ± 0	0 ± 0	4 ± 2	3 ± 1	4 ± 1
[MCPA]	2 ± 1	11 ± 2	17 ± 2	15 ± 2	25 ± 3
[MCPP]	1 ± 1	9 ± 1	10 ± 1	11 ± 1	13 ± 1
[2,4-D]	11 ± 2	68 ± 5	77 ± 6	71 ± 4	78 ± 5
[Dicamba]	0 ± 0	3 ± 1	5 ± 1	4 ± 1	7 ± 2

The highest overall mineralization efficiency was exhibited by isolates from the waste repository, whereas the lowest mineralization rates were observed for samples inoculated with microorganisms isolated from the river sludge. In general, the mineralization efficiency of the studied HILs by the isolated microbiota can be described by the following order: microbiota from the waste repository > microbiota from an agricultural runoff stream > microbiota from agricultural soil > microbiota from garden soil > microbiota from the river sludge.

The obtained results revealed that the mineralization rate of HILs with the [DecEtMor]^+^ cation was higher (0–31 %) compared to HILs with the [Dec_2_Mor]^+^ cation (0–20 %).

As a general trend, a moderate mineralization rate was observed for HILs with the [2,4-D] anion (9–31 %), whereas the mineralization observed for HILs with other anions was marginal (0–7 %). The influence of the herbicidal anion on the increase of mineralization can be described by the following order: [2,4-D]^−^ > [MCPA]^−^ > [MCPP]^−^ > [Dicamba]^−^.

Comparison of HILs and the corresponding reference compounds, which were used as either cations or anions during their synthesis, revealed that the mineralization efficiency of HILs was lower than that of the herbicides, especially 2,4-D (11–78 %) and higher than that of [Dec_2_Mor][Br] and [DecEtMor][Br] (0–4 %).

The OECD guidelines established that the studied compound must surpass the threshold mineralization efficiency of 60 % within 28 days in order to be considered as ‘readily biodegradable’. Taking this criterion into consideration, it can be concluded that the studied HILs cannot be classified as ‘readily biodegradable’. Among the reference compounds only 2,4-D was readily biodegraded by microbiota from the waste repository, agricultural soil, agricultural runoff stream and garden soil (78, 77, 71 and 68 %, accordingly).

The low mineralization rate of morpholinium-based ionic liquids corresponds well with the results presented by Pretti et al. (2011). The authors studied alkylmethylmorpholinium bromides and observed that the mineralization rate after 28 days was notably lower (>10 %) for cations with a long alkyl chain substituent (i.e. C10). Similar conclusions regarding the low biodegradability of methylmorpholinium halides with various side groups were reported by Neumann et al. (2014). The authors established that most of the studied morpolinium-based ionic liquids were not readily biodegradable, however the IL with cyanomethyl as a side group was susceptible to biotic hydrolysis, whereas the mineralization of IL with the (1-hydroxy)propyl side group reached 59 % after 41 days and was classified as ‘inherently biodegradable’. This suggests that certain modifications of the side chain may lead to an increase in mineralization rate and that morpholinium-based HILs may require a prolonged time to undergo ultimate biodegradation. Comparison of mineralization results with the analysis of surface active properties leads to a conclusion, that in this case the influence of the cation was similar to toxicity studies. The values for HILs with the [DecEtMor]^+^ cation and HILs with the [Dec_2_Mor]^+^ cation were similar, whereas the differences between the results obtained for HILs and commercial herbicides were notable. On the average, the mineralization of HILs with the [Dec_2_Mor]^+^ cation was lower by approximately 80 % compared to commercial herbicides in the acid form, however most of the results were very low, therefore this statement is only credible for HILs with the [2,4-D]^−^ anion.

The increased mineralization of HILs with the [2,4-D]^−^ anion may be attributed to an overall high biodegradability of the herbicidal moiety. As reported by Girardi et al. (2013), the mineralization of ^14/13^C-labelled 2,4-D in aqueous media reached 85 % after 28 days, which corresponds well with the results obtained in the framework of this study (high mineralization of 2,4-D was observed in all tests with the sole exception of microbiota from river sludge). Sanchis et al. (2014) reported low mineralization of MCPA (<25 % after 28 days), which is in accordance with the low ultimate biodegradation rate of MCPA by the isolated microbiota. No data regarding the mineralization of MCPP and Dicamba was found, however material safety data sheets of commercial products containing such herbicides indicate that they are not readily biodegradable.

### Evaluation of primary biodegradation of the studied HILs

An additional assessment of primary biodegradation of the studied HILs was carried out with the use of HPLC–MS. The obtained results were shown in Table 5. The values represent the loss of cationic or anionic moiety for a given IL, which was calculated based on the residual amount determined by HPLC–MS, expressed as a percentage relative to the initial amount of an ionic moiety for a given IL.

**Table 5:** Results of primary biodegradation tests for the studied HILs

Studied compound	Primary biodegradation (%)									
	Microbiota from river sludge		Microbiota from garden soil		Microbiota from an agricultural runoff stream		Microbiota from agricultural soil		Microbiota from a waste repository	
	Cation	Anion	Cation	Anion	Cation	Anion	Cation	Anion	Cation	Anion
[Dec2Mor][MCPA]	54 ± 9	0 ± 0	72 ± 11	0 ± 0	82 ± 12	40 ± 7	83 ± 9	37 ± 7	91 ± 6	35 ± 5
[Dec2Mor][MCPP]	61 ± 12	0 ± 0	71 ± 12	0 ± 0	81 ± 7	41 ± 5	82 ± 13	42 ± 5	88 ± 9	31 ± 4
[Dec2Mor][2,4-D]	58 ± 7	11 ± 2	74 ± 9	31 ± 6	90 ± 11	61 ± 9	92 ± 11	60 ± 9	94 ± 10	55 ± 9
[Dec2Mor][Dicamba]	55 ± 8	0 ± 0	77 ± 15	0 ± 0	81 ± 12	32 ± 5	79 ± 8	35 ± 5	86 ± 8	36 ± 8
[DecEtMor][MCPA]	42 ± 6	2 ± 1	64 ± 13	0 ± 0	79 ± 9	39 ± 6	78 ± 6	37 ± 8	81 ± 11	39 ± 7
[DecEtMor][MCPP]	41 ± 8	0 ± 0	60 ± 8	0 ± 0	78 ± 4	40 ± 8	74 ± 10	40 ± 6	84 ± 9	41 ± 8
[DecEtMor][2,4-D]	52 ± 11	9 ± 1	77 ± 9	25 ± 4	87 ± 14	60 ± 9	88 ± 11	60 ± 9	91 ± 7	51 ± 9
[DecEtMor][Dicamba]	38 ± 6	0 ± 0	56 ± 10	0 ± 0	77 ± 5	32 ± 6	75 ± 5	29 ± 4	83 ± 5	34 ± 6
[Dec2Mor][Br]	61 ± 10	x	86 ± 6	x	92 ± 10	x	91 ± 7	x	97 ± 10	x
[DecEtMor][Br]	48 ± 5	x	80 ± 7	x	88 ± 8	x	87 ± 9	x	93 ± 9	x
[MCPA]	x	26 ± 3	x	62 ± 9	x	66 ± 8	x	61 ± 9	x	74 ± 9
[MCPP]	x	21 ± 4	x	58 ± 8	x	62 ± 7	x	59 ± 8	x	71 ± 8
[2,4-D]	x	69 ± 7	x	96 ± 7	x	98 ± 6	x	95 ± 5	x	100 ± 6
[Dicamba]	x	13 ± 3	x	39 ± 4	x	52 ± 5	x	55 ± 5	x	64 ± 6

The results of primary biodegradation assessment are generally in accordance with the results of ultimate biodegradation tests regarding the ability of the studied isolates to biodegrade HILs. The highest biodegradation efficiency was observed for isolates from the waste repository as well as isolates from agricultural soil (depending on the type of IL and the corresponding cation and anion). The lowest biodegradation efficiency was observed for microbiota isolated from the river sludge (regardless of whether the cation or anion was analyzed). Hence, the primary biodegradation efficiency of the HILs decreased in the following order: microbiota from the waste repository ≥ microbiota from agricultural soil ≥ microbiota from an agricultural runoff stream > microbiota from garden soil > microbiota from the river sludge.

The primary biodegradation of the cations was higher for HILs with the [Dec_2_Mor]^+^ cation (54–94 %), whereas the results obtained for HILs with the [DecEtMor]^+^ cation were lower (38–91 %). The influence of the anion on the increase of primary biodegradation efficiency of the cation can be described by the following order: [2,4-D]^−^ > [MCPA]^−^ > [MCPP]^−^ > [Dicamba]^−^ .

The primary biodegradation efficiency of anions was highest among HILs with the [2,4-D]^−^ anion (0–60 %) and lowest for HILs with the [Dicamba]^−^ anion (0–36 %). The influence of the cation on the biodegradation of the anion varied depending on the structure of studied IL and the microorganisms used during the test, hence no unequivocal trends could be distinguished.

The primary biodegradation results obtained for the corresponding reference compounds, which were used as either cations or anions during the synthesis of HILs, suggest that the biodegradability of compounds used as cations was generally higher compared to compounds used as anions. The biodegradation efficiencies of [Dec_2_Mor][Br] and [DecEtMor][Br] were in the range of 61–97 % and 48–93 %, accordingly. The biodegradation efficiencies for MCPA, MCPP, 2,4-D and Dicamba were in the range of 26–74, 21–81, 39–100 and 13–64 %, accordingly.

The comparison of these results with those obtained for the cationic and anionic moieties of the studied HILs revealed two interesting implications. Firstly, the primary biodegradation of [Dec_2_Mor]^+^ and [DecEtMor]^+^ was only slightly altered when these compounds were used as cationic moieties in HILs and the values could be both decreased or increased compared to the biodegradability of reference compounds. Secondly, the primary biodegradation of MCPA, MCPP, 2,4-D and Dicamba was notably affected when these compounds were used as anionic moieties in HILs and the values decreased considerably in all cases compared to the biodegradability of reference compounds.

Comparison of primary biodegradation results with the analysis of surface active properties indicated that notable differences could only be observed when comparing results obtained for HILs with those obtained for herbicidal anions. On the average, the primary biodegradation of cations in HILs was lower by less than 10 % compared to cations in corresponding bromides, which is within the error margin. On the other hand, the primary biodegradation of anions in HILs with the [Dec_2_Mor]^+^ cation was lower by approximately 60 % compared to commercial herbicides in the acid form. Additionally, the cations were degraded as a preferential carbon source compared to the anions, however further studies are required in order to establish whether this phenomenon can be correlated with surface active properties. Primary biodegradation of HILs with tebuconazole- and propiconazole-based cations was recently reported in the study of Pernak et al. (2014b). The primary biodegradation of cations was only slightly lower compared to the precursor compounds (tebuconazole and propiconazole), whereas the biodegradation rate of anions was considerably lower compared to the precursor herbicides (MCPA, MCPP, 2,4-D and Dicamba), which is similar to the results obtained in the framework of this study. Other reports regarding primary biodegradation of herbicides suggest that all of the studied commercial crop protection agents may be efficiently transformed into subsequent metabolites. Ghoshdastidar and Tong (2013) observed a complete depletion of the initial concentration of 2,4-D after 12 days with subsequent formation of metabolites, which were also degraded (Ghoshdastidar and Tong 2013). Primary degradation of MCPA by *Enterobacter sp.* reported by Tan et al. (2013) was at 60 % after 28 days. This corresponds well with the results obtained in the framework of this study. On the other hand, Evangelista et al. (2010) observed a complete primary biodegradation of MCPA and MCPP by *Sphingomonas herbicidovorans* followed by the formation of biodegradable primary metabolites and poorly biodegradable secondary metabolites (Evangelista et al. 2010). This suggests that the primary biodegradation efficiency may be specie-dependant and explains why different results were obtained by microbiota from different niches. Interestingly, Gu et al. (2003) reported a high depletion of Dicamba concentration (approximately 95 % after 28 days), which is contrary to the obtained results (primary degradation of Dicamba was <70 % for all studied microbiota samples). However, it should be noted that in the experiments of Gu et al. (2003) the cultures were grown under methanogenic conditions, whereas the tests conducted in the framework of this study were carried out under aerobic conditions.

It is plausible that the cation was the preferred substrate due to the presence of long alkyl chains and the absence of chloride substitutes. Additionally, the relatively high primary biodegradation rate and low mineralization may suggest the occurrence of biotransformation processes, which lead to the formation of more toxic metabolites compared to the initial substrate, however more data is needed to verify this hypothesis.

## Conclusions

This study demonstrates that the toxicity and biodegradability of HILs in a given environment may vary, depending on the previous contact with contaminants (microbiota isolated from the waste repository exhibited higher resistance to toxicity and enhanced biodegradation efficiency compared to isolates from the river water sediment). It was also established that the structure and surface active properties of the cation may notably influence the toxicity and ultimate biodegradation of HILs. HILs with the more hydrophobic [Dec_2_Mor]^+^ cation exhibited better surface active properties, higher toxicity (EC_50_ values ranging from 45 to 222 mg L^−1^) and lower susceptibility to mineralization (0–20 %), whereas HILs with the more hydrophilic [DecEtMor]^+^ cation were less toxic (EC_50_ values ranging from 69 to 277 mg L^−1^) and mineralized more efficiently (0–31 %). Additionally, it was observed the cations were utilized as the preferential carbon source (41–97 %) during primary biodegradation processes compared to the anions (0–61 %). The influence of the anion on the studied properties of the HILs was also notable. The highest toxicity was displayed by HILs with the [MCPA]^−^ anion (EC_50_ values ranging from 45 to 190 mg L^−1^), whereas no apparent toxicity was observed for HILs with the [Dicamba]^−^ anion. The biodegradability tests indicate that HILs with the [2,4-D]^−^ anion were characterized by the highest susceptibility to both primary and ultimate biodegradation processes, whereas HILs incorporating other herbicidal anions displayed high resistance to mineralization.
